# Spatial complexity facilitates ordinal mapping with a novel symbol set

**DOI:** 10.1371/journal.pone.0230559

**Published:** 2020-03-26

**Authors:** Christine Podwysocki, Robert A. Reeve, Jacob M. Paul, Jason D. Forte

**Affiliations:** 1 Melbourne School of Psychological Sciences, University of Melbourne, Parkville, Victoria, Australia; 2 Department of Experimental Psychology, Helmholtz Institute, Utrecht University, Utrecht, The Netherlands; French National Center for Scientific Research (CNRS) & University of Lyon, FRANCE

## Abstract

The representation of number symbols is assumed to be unique, and not shared with other ordinal sequences. However, little research has examined if this is the case, or whether properties of symbols (such as spatial complexity) affect ordinal learning. Two studies were conducted to investigate if the property of spatial complexity affects learning ordinal sequences. In Study 1, 46 adults made a series of judgements about two novel symbol sets (Gibson and Sunúz). The goal was to find a novel symbol set that could be ordered by spatial complexity. In Study 2, 84 adults learned to order nine novel symbols (Sunúz) with a paired comparison task, judging which symbol was ‘larger’ (whereby the larger symbol became physically larger as feedback), and were then asked to rank the symbols. Participants were assigned to either a condition where there was a relationship between spatial complexity and symbol order, or a condition where there was a random relationship. Of interest was whether learning an ordered list of symbols would be facilitated by the spatial complexity of the novel symbols. Findings suggest spatial complexity affected learning ability, and that pairing spatial complexity with relational information can facilitate learning ordinal sequences. This suggests that the implicit cognitive representation of number may be a more general feature of ordinal lists, and not exclusive to number per se.

## Introduction

It is unclear if different ordinal sequences (e.g., numbers, letters, or novel symbols) share similar representational formats, or are represented/learned differently. This is an important issue, since it is claimed that ordinality is fundamental to numerical ability. Indeed, recent research has found that non-numerical ordered lists (i.e., letters of the alphabet) are represented in a similar manner to numerical information [1]. The authors interpreted this finding as implying ordinal lists share a common representational format. Nevertheless, it could be argued that familiarity with the position of letters in the alphabet lends itself to a representational format analogous to numerical representations. Potentially spurious claims about similar/different representational formats could be avoided by examining the ways unfamiliar ordinal symbol lists are represented and/or learned. To this end, this paper investigated if the property of spatial complexity could be used to learn the ordinality of a novel symbol set. Insofar as spatial complexity could be used for such an endeavour, it would suggest a common representational format supports ordinal lists more generally.

Ordinality refers to the location of an item in relation to other items in a sequence [2]. Ordinality and number are closely related, because when learning to count, children learn an ordered sequence of items [3]. For example, in the counting principles described by Gallistel and Gelman [4], children must learn that numerals always appear in the same order in a count (stable order principle), and that the numeral applied to the last item in a set represents the total number of items in the set (cardinality principle). However, it is not known whether these processes are unique to number, or whether they can be applied to ordered sets more generally. Research has shown that ordering non-numerical sequences in the first year of formal education (as measured by order judgements for everyday events and parents’ reports of their child's everyday ordering ability) predicted math ability one year later in young children [3], highlighting the importance of ordinal ability. Previous research has also suggested that ordinal sequences (such as numbers and letters of the alphabet) are served by a common representation [5–8], suggesting that ordinality and number are linked. Overall, these studies suggest that ordinality underlies number processing. However, it is not known whether ordinal sequences are learned/represented differently from these studies.

Researchers have examined the representation of ordinal sequences using letters of the alphabet, or other ordinal lists, such as months of the calendar year [1,9–13]. For example, Podwysocki, Reeve, and Forte [1] compared judgements of numerical stimuli and letter stimuli on a number-to-position task for numbers, and an alphabet-to-position task for letters. Specifically, participants were shown a horizontal line anchored by 1 to 26 for numbers, and A to Z for letters, and were required to estimate the location of a target number or letter respectively. The authors found that numbers and letters both produced linear patterns of response for these tasks, and that the patterns of non-linear responses were also similar for the number and letter stimuli. These findings are supported by researchers who have found similar response patterns for numbers and letters on a spatial mapping task [9,13]. However, see [14] for an alternative finding with brain imaging techniques. Indeed, a possible issue using letters to make inferences about numerical representations is that letters of the alphabet and numbers are often learned in a similar way (i.e., 1, 2, 3, and A, B, C are learned first in the number and letter sequences), and at a similar developmental stage, which may result in a similar representation for number and letter stimuli, and explain the results from studies comparing number and letter representations. One way to overcome this interpretive limitation is to use novel symbols.

Novel symbols are often used in research to study ordinal list learning. For example, Lyons and Ansari [15] used novel visual shapes to test the cerebral basis of mapping non-symbolic number onto abstract symbols. Lyons and Beilock [16] also used the novel visual shapes to test whether working memory capacity affects the ability to infer ordinal relationships between symbols. The Gibson symbol set (see [17] for an image of the Gibson symbols) [18–20] is also widely used to test magnitude relations and representations in neurologically healthy adults [17,21] and in adults with math learning disabilities [22,23]. However, a potential issue with the Gibson symbols is that they were created by transforming letters of the Roman alphabet [20]. Another symbol set that has been used more recently that was not made from letters of the alphabet are the fictional numbers from the script of the Sunúz (see [24] for an image of the Sunúz symbols), from the fantasy world of *Tékumel*: *Empire of the Petal Throne* [25]. Research by Merkley et al. has used the Sunúz symbol set to test the role of magnitude information and ordinality in number representations in adults to avoid existing knowledge of ordinality in Hindu-Arabic numerals [24,26].

While novel symbols have not been used to test whether the representation that underlies number is common to ordinal lists, they offer a means of avoiding the over-learned and over-familiar Hindu-Arabic numerals, letters of the Roman alphabet, or other common everyday symbol lists [27]. They also allow for an examination of whether some property of the symbols (such as spatial complexity) affects ordinal list learning, an impossibility with more common everyday symbols as they contain information from prior learning. For novel symbols to be useful for this task, it would be beneficial if they could be ordered by the property of spatial complexity (where indices of ‘complexity’ can include the number of parts, total edge length, the GIF compression metrics, or the density of a symbol [28, 29]). It may be possible to use spatial complexity as an indicator of ordinality because there is a link between the numerosity a symbol represents and the spatial complexity of the symbol in a range of different cultures [30–32]. For Chinese numerals, ‘one’ is one horizontal line, ‘two’ is two horizontal lines, and ‘three’ is three horizontal lines. For Roman numerals, ‘one’ is one vertical line, ‘two’ is two vertical lines, and ‘three’ is three vertical lines. For Hindu-Arabic numerals, one is a single line, and the numerals ‘two’ and ‘three’ may derive from two or three horizontal lines that became linked together when they were deformed by handwriting [33]. This observation suggests that ordering a novel symbol set by spatial complexity could enhance ordinal symbol mapping.

One method for training an ordinal list with novel symbols (and used in this research) is a paired comparison relational learning task. A participant would learn the order of a list of symbols by making ‘greater than’ judgements for a series of novel symbols pairs. With a series of paired comparison judgment trials, the mental representation of the ordered list of symbols could be tested. This would allow for an assessment of whether the ordinal representation of novel symbols resembles the ordinal nature of numerical representations.

The studies reported herein investigated whether the spatial complexity of a list of novel symbols would facilitate learning an ordinal sequence. Study 1 evaluated whether the Gibson and Sunúz symbol sets could be ordered by complexity by asking participants to rank the symbols according to their numerical value (e.g., ‘which symbol is best suited to represent a small number?’) and their complexity (e.g., ‘which symbol do you think is the most complex?’). This was done so a condition could be created that randomised the spatial complexity of the novel symbols. Study 2 tested the hypothesis that learning a novel symbol set ordered by spatial complexity would facilitate ordinal list learning when compared to learning a novel symbol list ordered by random complexity. It was expected that learning a list of novel symbols would be facilitated when symbols were ordered by spatial complexity, compared with spatial complexity not being an indicator of ordinality.

## Study 1

The goal of Study 1 was to select a novel symbol set where symbols were consistent in terms of perceived complexity rankings but did not evoke other symbol sets (such as Hindu-Arabic digits or letters from the alphabet). Nine Gibson symbols and nine Sunúz symbols were selected for testing. To test the novel symbol sets, participants were required to order each symbol set from small to large, and from simple to complex. Participants also mapped the symbols directly onto a list of symbolic (i.e., Hindu-Arabic digits) and non-symbolic (i.e., dot arrays) number stimuli. The purpose was to identify which novel symbol set had the most consistent pattern between perceived numerosity and complexity judgements (i.e., that participants would reliably rate specific symbols as ‘large’, ‘small’, ‘complex’, and ‘simple’). In summary, the novel symbol set that is determined to have the most consistent perceived complexity rankings would make a good candidate for testing in Study 2. Finally, to ensure participants were ranking symbols according to complexity and not another property, an objective complexity analysis was also conducted.

### Method

#### Participants

Forty-six (*M* = 19.34 years, *SD* = 2.99 years; 24 males, 22 females) undergraduate students from an Australian university participated in the research for course credit. All participants had normal or corrected-to-normal vision. Written and informed consent was obtained by asking participants to read a plain language statement and then sign a consent form. All procedures involved were approved by, and in accordance with, the University of Melbourne Human Ethics Advisory Group (HREC number 1441499).

**Apparatus.** Stimuli were created on a Dell OptiPlex 9020 computer running Ubuntu 12.04 with MATLAB software and Psychophysics Toolbox routines [34–36] and displayed on a 23-inch Dell E2314H LED monitor operating at a spatial resolution of 1,920 by 1,080 pixels at a refresh rate of 60Hz.

#### Stimuli

Two sets of novel symbols were chosen to test in this study. The first symbol set were a subset of Gibson figures. The second symbol set were fictional numbers of the script of the Sunúz from the fantasy world of *Tékumel*: *Empire of the Petal Throne*.

The first task required participants to make four sets of judgments regarding the Gibson symbols and the Sunúz symbols. Symbols were presented on the screen in a circular arrangement (which was the same for each participant, and the symbols were arranged pseudo-randomly). Judgements included: a) ‘which symbol you would choose to represent a small number?’; b) ‘which symbol you would choose to represent a large number?’; c) ‘which symbol do you think is the simplest?’; and d) ‘which symbol do you think is the most complex?’. At a viewing distance of 60cm, the symbols were 2 degrees of a visual angle high (1 degree = 1cm). Instructions remained on the screen for the duration of the testing period. All stimuli were presented in black on a white background. A small black circle appeared around each symbol as the cursor moved over it. The cursor returned to the centre of the screen after every trial to re-orient participants. Each time participants clicked a symbol, it disappeared, resulting in one fewer symbol to select from. This process continued until all symbols were removed from the screen. For all tasks, the Gibson symbols were presented first, followed by the Sunúz symbols for consistency. The second task required participants to make four different sets of mapping judgements regarding the Gibson and the Sunúz symbols. The stimuli were presented in an identical format to the previous task. Participants were asked to choose the symbol that was most similar to the number in the middle of the circle. These questions were presented in order (from 1 to 9) and reverse order (from 9 to 1) for the symbolic (i.e., Hindu-Arabic digits) and non-symbolic (i.e., dot arrays) number sets.

#### Procedure

Testing was conducted in a quiet testing space. The study began with instructions informing participants that they would be asked to click some symbols, and that there were no correct or incorrect answers. The first task was the symbol judgment task, where participants were presented with a series of questions for both symbol sets. There were four different questions, with one trial for each of the nine stimuli, with a total of 72 trials. The second task was the symbol mapping task, where participants were presented with mapping questions for both symbol sets. There were four mapping questions, with one trial for each of the nine stimuli, resulting in 72 trials.

### Results and discussion

**Fig 1:**
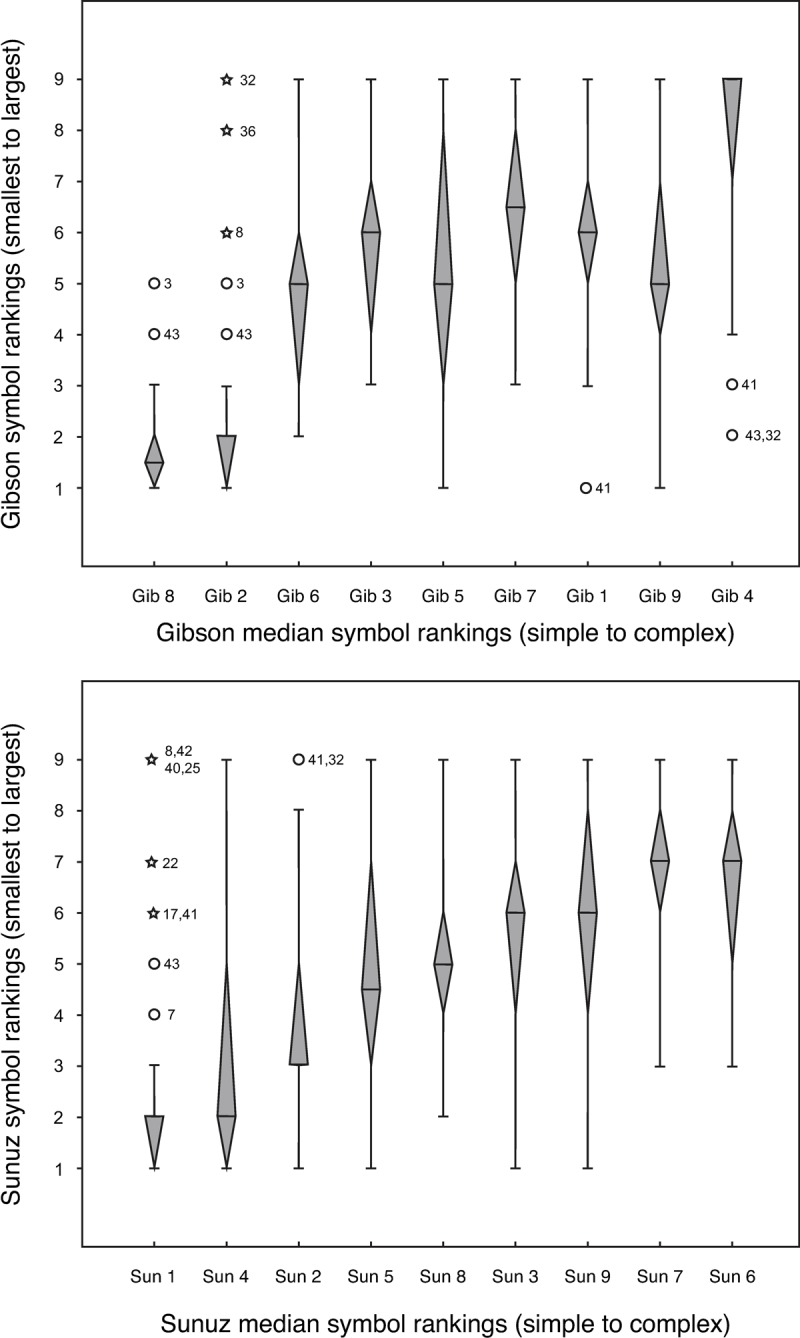
Box plots displaying median small to large symbol rankings (y-axis) ordered by the median simple to complex ranking (x-axis) for the Gibson symbols (top) and Sunúz symbols (bottom). Regular outliers are denoted by open circles and represent 1.5 times the interquartile range. ‘Extreme’ outliers are denoted by stars and represent 3 times the interquartile range. The specific numbers next to outliers correspond to individual participants.

To determine whether the novel symbols had a consistent relationship between perceived number rakings and complexity rankings, the median rankings were calculated for each task for both symbol sets. Median rankings were used instead of mean rankings, since outliers were present in these data. Fig 1 displays box plots of the small to large number rankings (y-axis) ordered by the median simple to complex rankings (x-axis) for the Gibson (top) and Sunúz (bottom) symbols, respectively.

In these box plots, an ascending monotonic relationship between the simple to complex and smallest to largest rankings would suggest a relationship between number and complexity. The Gibson and Sunúz symbol sets have a similar relationship between perceived complexity and number, displaying an ascending relationship between number and complexity rankings. Also, for both symbol sets, there is a consistent trend where the symbols ranked as simpler are also ranked as a smaller number and the symbols ranked as more complex are also ranked as a larger number. This indicates that either symbol set could be used for the next study. However, the boxplots show that the Sunúz symbols had a clearer monotonic relationship between complexity rankings and fewer outliers than the Gibson symbols. The R^2^ line fit from the median values is 0.95 for the Sunúz symbols, and 0.67 for the Gibson symbols, indicating a stronger relationship between perceived complexity and number for the Sunúz symbols. Furthermore, because many of the Gibson symbols resemble common everyday symbols and were created by transforming letters of the alphabet [15], it was decided that the Sunúz symbols were better suited to the purpose of Study 2.

To determine whether participants were ordering the Sunúz symbols by their complexity, the subjective ordering of complexity provided by participants was compared with six other quantitative measures of complexity. Since there are multiple conceptual and mathematical definitions of ‘complexity’, this study chose to examine two entropy-based measures (e.g., total absolute curvature [37,38] and number of concavities), two image-based measures (e.g., symbol perimeter (mm) and symbol area (mm^2^)), and two algorithmic-based measures (e.g., GIF compression [29] and Block Decomposition Method (BDM) [39]).

Total absolute curvature quantifies the inflection points of a curve [40], in this case the geometry of each symbol. Total absolute curvature (*T*) is calculated as $T=\frac{1}{2\pi }\int_{L}^{0}\left|\kappa \left(l\right)\right|dl$, for a curve length (*l*), following a curvature function (*κ*(*l*)), over the total length of the curved profile (*L*). Changes in the sign of curvature profile suggest concave regions within each symbol, so the number of concavities in each symbol were also measured as an index of total absolute curvature (calculated using an efficient Fourier transform implementation [41]). The perimeter and area of each symbol were calculated using Inkscape (Measure Path extension). GIF compression is measured as the size of each symbol’s image file (KB). GIF images are a lossless compressed format based on the Lempel-Ziv-Welch compression algorithm [42,43] and as such provide a measure of sub-pattern redundancy. BDM approximates local estimates of algorithmic complexity, which derive from redundancy measures of higher order entropy. BDM was calculated here using the implementation described in [39]. Spearman’s rank order correlations were calculated between the symbol orders determined by each of the complexity measures (Table 1).

**Table 1:** Spearman’s rank order correlations between different measures of complexity.

	(1)	(2)	(3)	(4)	(5)	(6)	(7)
(1) Subjective	1						
(2) Total absolute curvature	-.20	1					
(3) Concavities	.95***	-.23	1				
(4) Perimeter (mm)	.90**	-.05	.75*	1			
(5) Area (mm^2^)	-.07	.77*	-.12	.17	1		
(6) GIF compression	.82*	-.08	.73*	.78*	.22	1	
(7) BDM	.67	-.30	.83**	.30	-.30	.42	1

*** *p* < .001,

** *p* < .01,

* *p* < .05.

As can be seen in Table 1, the subjective ordering of complexity provided by participants was highly correlated with entropy, image and algorithmic measures of complexity. The total number of concavities (i.e., inflection points in each symbol’s curvature profile) was highly correlated with subjective complexity rankings, suggesting participants were sensitive to the entropy-based complexity of the symbols. Participants also judged symbols with a greater total image perimeter and less redundancy in repeatable sub-patterns (i.e., GIF compression rate) as being more complex.

Examining correlations between the quantitative measures of complexity also provide useful information about the nature of the Sunúz symbol set itself. For example, higher total absolute curvature scores correlated with greater total symbol area, suggesting a limit on the entropic complexity of symbols as a function of their size. Symbols with a higher number of concave regions correlated with a longer perimeter, and greater algorithmic complexity, suggesting symbols with greater changes in curvature are more difficult to algorithmically reproduce and simplify (i.e., less repetition of sub-patterns). Lastly, symbols with a longer perimeter were also correlated with larger GIF file sizes (i.e., less able to compress).

## Study 2

The aim of Study 2 was to determine if learning a set of novel symbols would be facilitated by associating list position with spatial complexity. Prior research has claimed that the symbols are mostly abstract [33], implying that the format of the symbols is irrelevant. However, research has not yet tested whether the underlying spatial properties in symbols affect learning ordinal relationships. The aim of this study was to test if spatial complexity could facilitate learning a novel symbol set. If it is found that spatial complexity enhances learning novel symbols, it would suggest that ordered lists can be learned with only relational information and the property of spatial complexity.

Participants were presented with pairs of Sunúz symbols and were required to learn the order of the symbols through relational information (i.e., participants were asked ‘which symbol do you think is the largest?’). Participants were provided with visual feedback (the symbols either increasing or decreasing in size to indicate whether the symbol is ‘larger’ or ‘smaller’, respectively). To test if spatial complexity facilitates learning the novel symbol list, participants were pseudo-randomly allocated to one of two learning conditions. Participants in the first group learned the symbols ordered by the median perceived complexity rankings from Study 1. Participants in the second condition learned the symbols in a random complexity order, where the spatial complexity of the symbols was not associated with the ordinal position of the symbol. Participants were then shown symbols in a circular arrangement and clicked on the symbols according to the order they learned during the training phase. If participants in the spatial complexity group outperformed participants in the random complexity group, then this would suggest properties such as spatial complexity can act as a mediating construct when learning internal ordinal representations.

### Method

#### Participants

Eighty-four (*M* = 19.05 years, *SD* = 2.72 years; 30 males, 54 females) undergraduate students from an Australian university participated in the research for course credit. All participants had normal or corrected-to-normal vision. Written and informed consent was obtained by asking participants to read a plain language statement and then sign a consent form. All procedures involved were approved by, and in accordance with, the University of Melbourne Human Ethics Advisory Group (HREC number 1441499).

#### Apparatus

The apparatus was the same as used in Study 1.

#### Stimuli

The nine Sunúz symbols were used in Study 2. There were two groups which participants were pseudo-randomly allocated to. In the first group, the symbols were ordered according to their spatial complexity, which was determined by the median complexity rankings in Study 1. In the case of a tied median ranking, the mean was used to decide the order. In the second group, the symbols were ordered randomly, where both halves of the symbol list were matched for complexity (i.e., both halves were equally complex).

Study 2 had two phases. The first phase presented participants with pairs of Sunúz symbols (the paired comparison task), and they were asked to judge which symbol denoted a ‘larger’ value to learn the order of the symbols (where the order of the symbols refers to the order of the ‘larger’ judgements). The second phase presented participants with the nine symbols on the screen in a circular arrangement (which was the same for each participant) to assess how well participants learned the order of the symbols in the training phase. Participants ranked the symbols from smallest to largest, corresponding to the order they learned during training. Each time participants clicked a symbol, it disappeared from the screen, resulting in one fewer symbol to select from. This continued until all symbols were removed from the screen.

At a viewing distance of 60cm, the symbols were 2 degrees high. As feedback, the ‘larger’ symbol appeared physically larger on the screen (4 degrees high), and the ‘smaller’ symbol appeared physically smaller on the screen (1 degree high). The cursor returned to the centre of the screen after every trial to re-orient participants. Symbols remained on the screen until a decision was made.

#### Procedure

Testing was conducted in a quiet testing space. The study began with instructions informing participants that they would be asked to determine the numerical order of some symbols in a paired comparison task. Participants were first told that two unfamiliar symbols would appear on the screen, and that one symbol was ‘larger’ than the other one. Participants were then instructed that they were to learn the order of the symbols, and to click on the symbol that was ‘larger’. Participants were told that feedback would be provided by the size of the symbols changing to reveal the larger symbol. Each trial presented a pair of symbols, where every symbol was paired with every other symbol excluding itself in both left-to-right and right-to-left orientations. There was a 500ms gap between each trial. There were 6 blocks consisting of 72 feedback trials each, with a total of 432 trials.

After each training block, participants were shown a new instruction screen, which informed them that they were now required to order all symbols from smallest to largest. This task was designed to assess global symbol list learning. Participants were then presented with the symbols in a circular arrangement and ranked the symbols according to the order in which they were learned during training.

### Results and discussion

**Fig 2:**
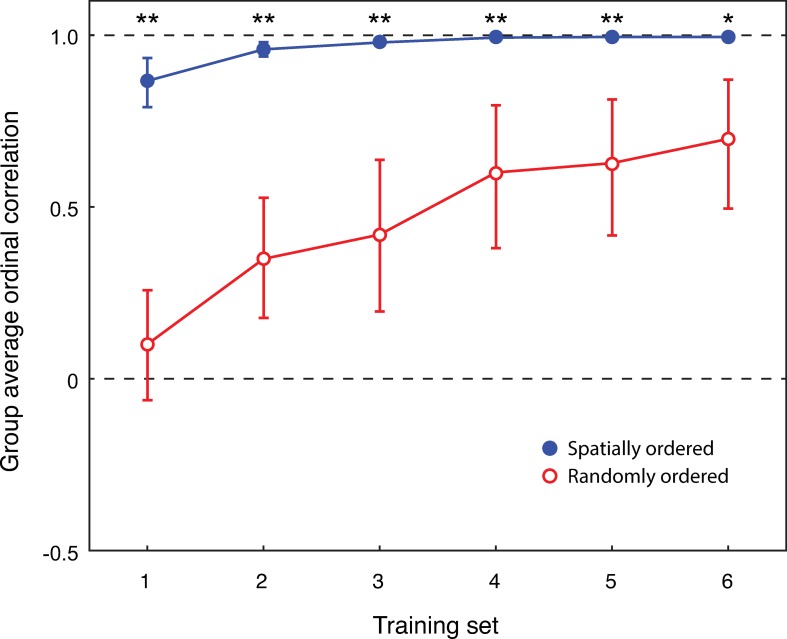
Group average Spearman’s rank order correlations (y-axis) for both the spatially ordered group (filled blue circle) and the randomly ordered group (open red circle), as a function of training session (x-axis). The error bars represent the 95% CI. The dashed lines are for correlations of 0 (no correlation) and 1.0 (perfect correlation). Two stars is significance at the *p* < .01 level, and one star is significance at the *p* < .05 level.

To determine whether there was a difference between the spatial complexity group and the random complexity group in their ordinal rankings of the symbols, the mean group Spearman’s rank order correlations were calculated for both groups for each training session (Fig 2). Spearman’s rank order correlations are a nonparametric measure of rank correlation which assesses how well the relationship between two different variables can be described using a monotonic function. Spearman’s rank order correlations were chosen for Study 2 because they provide a simple way of assessing whether the correlated spatial complexity and random complexity groups displayed a difference in their ability to order the symbols. In the context of this study, a Spearman’s rank order correlation of 1.0 would indicate a perfect ordering of the symbols, 0 would indicate a random ordering of the symbols, and -1.0 would indicate a reverse ordering of the symbols.

Several participants did not complete the six training blocks within the 25 minute time allocated for the session (*n* = 2 in the spatial complexity group, and *n* = 8 in the random complexity group). For these participants, the last correlation they achieved was used to replace missing blocks. Correlations were calculated for each participant between their ordering in each block and the ‘correct’ order. Examining the means and 95% confidence intervals (CIs), the spatial complexity group (blue) outperformed the random complexity group (red). The spatial complexity group learned the correct order quickly and consistently; block 1 = 0.87 [0.80, 0.94], block 2 = 0.96 [0.94, 0.98], block 3 = 0.98 [0.97, 0.99], block 4 = 0.99 [0.99, 1.00], block 5 = 1.00 [0.99, 1.00], and block 6 = 1.00 [0.99, 1.00], often achieving near perfect performance from the second training block, with an average correlation of 1.00 by the sixth training block. In contrast, the random complexity group displayed much slower learning; block 1 = 0.10 [-0.05, 0.26], block 2 = 0.35 [0.18, 0.53], block 3 = 0.42 [0.20, 0.64], block 4 = 0.60 [0.38, 0.82], block 5 = 0.63 [0.42, 0.83], and block 6 = 0.70 [0.49, 0.90], achieving an average correlation of only 0.70 by the sixth training block.

**Fig 3:**
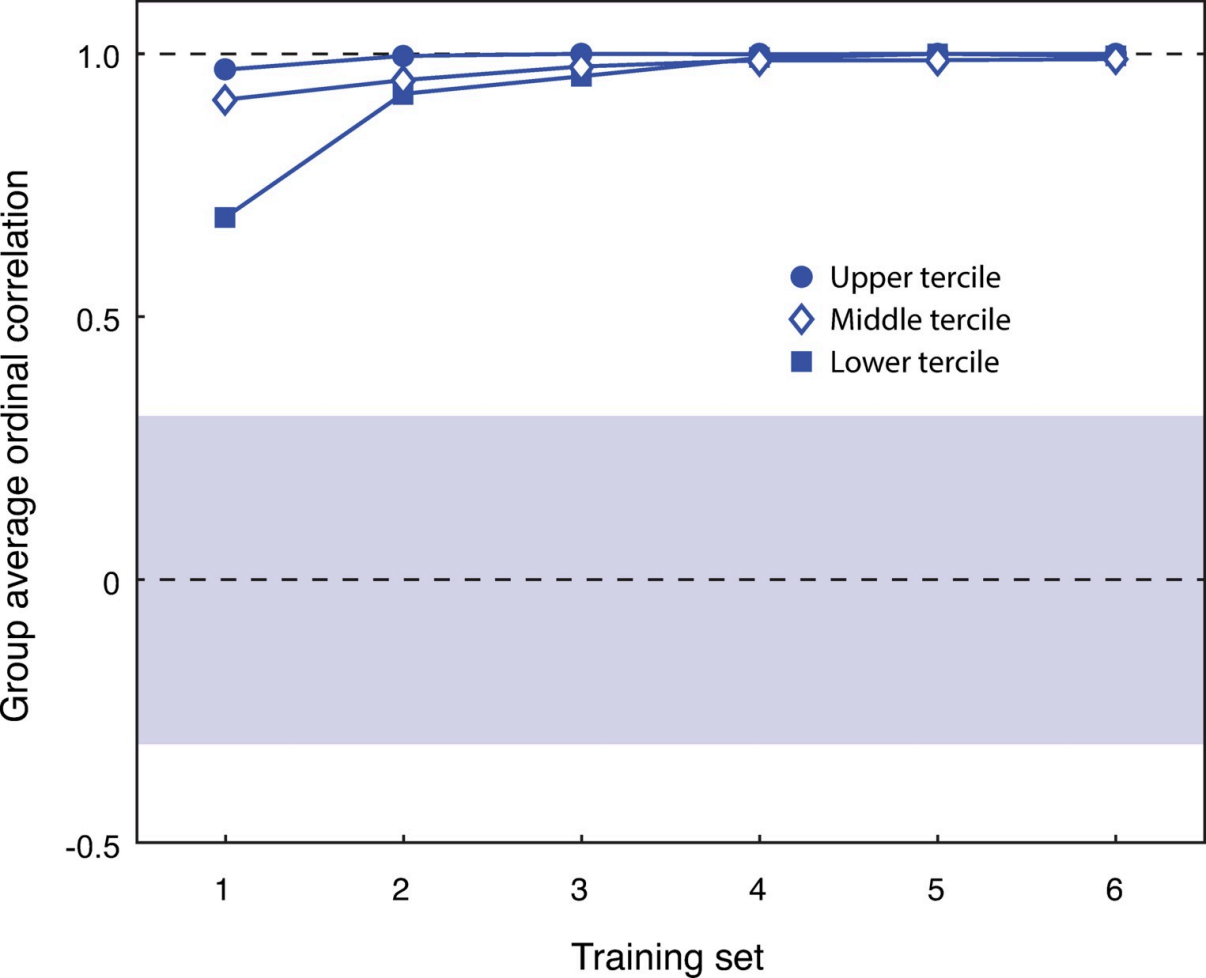
Spatial complexity group split into fastest (filled circle), middle (open diamond), and slowest (filled square) learning terciles as a function of training session (x-axis) by mean Spearman rank order correlation (y-axis). The blue bar represents the bootstrap 95% CI for the spatially ordered group. The dashed lines are for correlations of 0 (no correlation) and 1.0 (perfect correlation).

**Fig 4:**
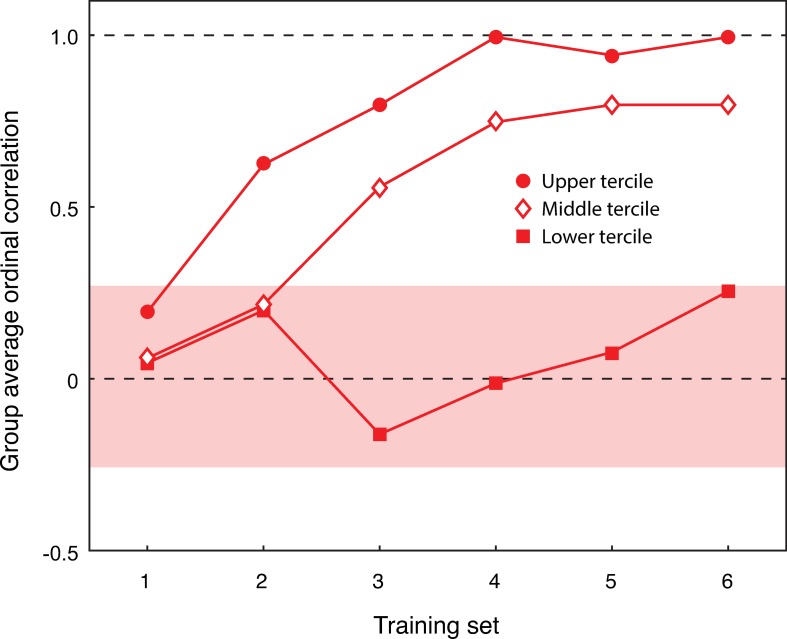
Random complexity group split into fastest (filled circle), middle (open diamond), and slowest (filled square) learning terciles as a function of training session (x-axis) by mean Spearman rank order correlation (y-axis). The red bar represents the bootstrap 95% CI for the spatially ordered group. The dashed lines are for correlations of 0 (no correlation) and 1.0 (perfect correlation).

The error bars for the two groups revealed there is minimal variation in the rate of learning for the spatially ordered group. There is some variation in the first training block, which reduces after the second training block. The random complexity group displayed more variation, with large error bars for every training session. The variability in these data suggest that it would be useful to split the two groups into different levels of learning performance, to better examine the differences in learning within the spatially ordered group (Fig 3), and the random complexity group (Fig 4). The 95% CIs were computed from the average correlations of each group using a bootstrapping method (random sampling with replacement). For the spatial complexity group, the bootstrap 95% CI was [-0.31, 0.31]. For the random complexity group, the bootstrap 95% CI was [-0.27, 0.26].

The learning profiles for the spatial complexity group are similar in structure. The slowest learning tercile display lower performance in the first training block compared to the middle and fastest terciles. However, by the second training block, the performance for all three terciles is comparable. This difference could possibly be explained by the fact that several of the symbols received similar complexity rankings particularly towards the middle of the symbol list in Study 1. The bootstrap 95% CI for the spatially ordered group confirms that these data cannot be explained by chance, and that the participants successfully learned the correct order of the novel symbols in this condition.

In comparison, in the random complexity group, the learning profiles of the fastest, middle, and slowest learning terciles were different from each other. While the fastest and the middle learning terciles are comparable (with the middle learning tercile having a similar format, albeit slower), the slowest learning tercile shows little evidence of having learned the correct order of the symbols. The bootstrap 95% CI for the random complexity group confirms this, and suggests that performance of this tercile is not distinguishable from chance performance. Indeed, the final correlation achieved for the slowest learning tercile is less than 0.26. While the fastest and middle terciles for the random complexity group display some evidence of successfully learning the correct order, the slowest learning tercile failed to learn the correct order of the novel symbols.

## General discussion

The aim of this research was to determine whether learning a set of novel symbols would be facilitated if symbol list order was associated with the underlying property of spatial complexity. This was done to investigate the ordinal relations that may underlie number representations, and to determine if ordinal lists are served by a common cognitive representation. Specifically, the goal was to determine if ordering symbols according to spatial complexity improves list learning ability. The key finding of this research was that participants in the spatial complexity group learned the order of the new symbols better than participants in the random complexity group. This finding supports the view that underlying information contained in symbols may provide relational information that influences learning internal representations of ordered lists.

This study found that participants who learned the novel symbol set where list order was associated with spatial complexity often learned the correct order in the first training block, and performance thereafter was almost at ceiling level for most participants. In comparison, in the random complexity condition, learning the relationships between novel symbols was more difficult. In this condition, most participants never reached ceiling performance, while many also failed to score above chance level performance after the six training blocks. This suggests that participants can easily use the spatial complexity of novel symbols to assist them in making paired comparison judgements, and to reconstruct the global order of the novel symbol list. A possible caveat is that it is not entirely clear whether symbol complexity facilitates learning, or whether randomised spatial complexity inhibits list learning. In either case, it seems there is a relationship between learning an ordered list of novel symbols and the spatial complexity of those novel symbols.

There are various possible reasons why some participants failed to learn the randomly ordered novel symbols. From looking at the fastest and middle learning terciles, it can be seen that learning this symbol list was not impossible, because some participants still learned the correct order of this list even without the spatial complexity cue. Therefore, it seems that some participants may have difficulty learning a list of novel symbols, such that six training blocks was not enough time to learn the correct order. A possibility that could be investigated in future research is that participants could be learning ‘chunks’ of the correct order, but failing to learn other parts of the sequence, leading to an overall low correlation. Another possibility is that participants in the random complexity group could have had an expectation that the novel symbols would be ordered by spatial complexity from some of the feedback provided, and when this was not the case, learning the correct order of these symbols became difficult. It is important to note that the sample of Study 2 were educated university students. If this task was given to younger children with less education, then performance in the random condition could be even worse, perhaps with no participants learning the correct order. Alternatively, it is also possible that young children would be better at this task, since they would have no prior expectations regarding the order of the novel symbols.

Symbols are often thought to be mostly abstract objects [33]. However, if this was the case, then there should not have been any difference between the two conditions, especially since this study accounted for differences in recognition of the symbols by including the random complexity condition. If participants were not attending to the underlying spatial properties of the novel symbols when learning the ordinal lists, there should have been no advantage for the spatial complexity condition over the random complexity condition. This casts some doubt on the claim that numerical symbols are entirely abstract, and suggests the spatial complexity of number symbols and the relational information they convey should be considered when studying number representations. However, it could also be the case that spatial complexity was used as a cue because the symbols were unknown, and that over time, spatial complexity has less of an impact. Overall, this research suggests that when spatial complexity is paired with relational information, this is sufficient to create an ordinal representation akin to the ‘mental number line’ (MNL) [33].

The finding that the property of spatial complexity had an effect on learning has implications for thinking about how ordinal lists are represented. In relation to the MNL metaphor, which places large emphasis on the numerical values of number symbols (cardinality), the findings of this study suggest that ordinality and the underlying spatial properties of symbols should be highlighted more when learning numbers, because number symbols may provide information which assists with learning. For instance, the ease with which the first three numbers in a range of cultures are learned may have something to do with their spatial complexity [30–32], which could provide a framework for how other numbers are mentally represented. This is observation is highlighted by looking at the correlations for Study 2. While the spatial complexity group performed well overall, the random complexity group displayed unpredictable responses, with some participants failing to learn the correct order, even after six training blocks. This may be because the random complexity group had no consistent spatial complexity information in the symbols to assist them learning the correct order. However, it is of course also possible that this could be related to these early numbers falling in the subitizing range, and the fact that the focus of attention in working memory contains approximately three to four elements [44].

This research found that ordering symbols by spatial complexity enhanced novel symbol learning. This suggests that learning number symbols could also involve this relational component. The property of spatial complexity may also be a relevant dimension to consider in creating number representations, because it strongly relates to how ordered sequences or patterns were generated in this study. As mentioned above, relational information and spatial complexity seem to be sufficient to derive an internal ordered representation of a novel symbol set. This broadly relates to the role of perceptual features in numerical processing [45], and also the idea that numerical ordering processes occur relatively early in development [46]. Furthermore, since the spatial complexity of a symbol can only be understood in relation to other symbols, discussing number representations without considering the relational structure of symbols means important factors of number representations are likely being overlooked. Indeed, research is beginning to highlight the importance of symbolic number competence and knowledge of ordinality with formal math achievement [47], and this study adds to this body of research by suggesting that underlying spatial properties of symbols (e.g., spatial complexity) may be important to consider when thinking about mental number representations.

Beyond symbols containing relational information, an important point to make is that the perceived spatial complexity of the symbols was fairly consistent across all participants in Study 1. When asked to order a new set of symbols by complexity, most participants ordered the symbols in the exact same way, despite not being told what to use to perform these complexity rankings. This suggests that people may spontaneously order lists by spatial complexity, which may further explain why the random complexity group performed so poorly, because the relational structure of the symbols was incongruent with the feedback provided. Extending this finding to number, it suggests that the Hindu-Arabic numerals may be a difficult symbol set to learn, especially for those with maths difficulties such as dyscalculia [48]. For example, if a child is struggling to learn number, they may have a poor knowledge of numerical symbols, similar to the random complexity group in this study. From these findings, it seems that it may be beneficial to emphasise the spatial properties of Hindu-Arabic numerical symbols more during number learning in an educational setting.

A limitation of this study is that it did not test whether the novel symbols in this study acquired mathematical properties, which may limit the inferences that can be made from this research. The next step for future research would be to create mathematical relationships between these symbols (such as with addition or multiplication tasks), to examine whether these symbols can take on the computation properties of number. It would also be interesting to ask participants to complete a ‘symbol-to-position task’ (similar to a number-to-position task but with novel symbols) for these novel symbols for both the spatial complexity and random complexity conditions. If it was found that the spatial complexity condition produced more accurate responses, then this would suggest that spatial complexity may be used when completing numerical tasks, which means metaphors of number representation should take this into account. Another interesting line of research would be to study post-hoc whether differences in the paired comparison task predict differences in overall ordinal ranking. For now, this research can be interpreted as a ‘proof of concept’ that the spatial complexity of symbols can influence the learning of ordinal lists of symbols, which most likely also underlies number representations. This also suggests that the MNL metaphor may be better thought of as a ‘mental ordinal line’ that underlies ordinal lists more generally. This ties into previous research [49] that suggests attainment and use of ‘knowledge structures’ underpin numerical representation; and other research [50] that suggests a more generalised MNL could be built in working memory irrespective of the items being studied (e.g., [50] used the words for fruits and vegetables as stimuli in a categorization task).

Overall, this research shows that underlying spatial properties of symbols (such as spatial complexity) may be important for learning internal ordinal relationships between symbols in a list. By asking participants to learn a novel symbol set, this study was able to manipulate learning in adult participants. This research found that when spatial complexity was linked with list order, this aided learning. In this case, spatial complexity was used as a proxy for ordinal relationships between symbols to help participants learn the correct order of the symbols. This suggests that the spatial complexity of symbols should be accounted for in future models of number representation, and that research should consider the relational information between number symbols. Since an ordinal representation was created with novel symbols, it seems likely that a more general cognitive representation underlies ordinal lists.
